# Evaluating Biochar Impact on Topramezone Adsorption Behavior on Soil under No-Tillage and Rotary Tillage Treatments: Isotherms and Kinetics

**DOI:** 10.3390/ijerph16245034

**Published:** 2019-12-10

**Authors:** Jean Yves Uwamungu, Obemah David Nartey, Fasilate Uwimpaye, Wenxu Dong, Chunsheng Hu

**Affiliations:** 1 Key Laboratory of Agricultural Water Resources, Center for Agricultural Resources Research, Institute of Genetics and Developmental Biology, Chinese Academy of Sciences, Shijiazhuang 050021, China; 2 University of Chinese Academy of Sciences, Beijing 100049, China; 3 State Key Laboratory of Soil and Sustainable Agriculture, Institute of Soil Sciences, Chinese Academy of Sciences, Nanjing 210008, China

**Keywords:** topramezone, adsorption, kinetics, isotherm, biochar, tillage

## Abstract

The evaluation of biochar application on the adsorption behavior of topramezone on soil under no-tillage (NT) and rotary tillage treatments (RT) has been assessed. Fourier Transform Infra-Red Spectrometry (FTIR), scanning electron microscopy (SEM), and Brunauer–Emmett–Teller) (BET) were used for the biochar characterization. Batch experiments were carried out in a laboratory to assess the adsorption of topramezone on soil through equilibrium and kinetic modeling under biochar addition. The clay content has been found to be higher under NT (18.24 ± 0.01) than under RT (15.91 ± 0.02). The total organic carbon was higher under NT. The topramezone adsorption equilibrium reached after 8 and 12 h, for NT and RT, respectively. The kinetic and thermodynamic analyses showed the adsorption under both treatments matched with pseudo-second-order kinetic and Langmuir models, respectively. After biochar addition, the pesticide adsorption capacity (40 < 25 < 15 °C) increased with decreasing temperature suggesting an exothermic adsorption process while negative values of Gibbs free energy (ΔG); −1848.07 and −366.531 J mol^−1^; for the soil under NT and RT at 25 °C, respectively, indicated spontaneous adsorption. Negative entropy values (ΔS); −21.92 and −78.296 J mol^−1^K^−1^, for NT and RT, respectively, explained a decreased randomness process. The enthalpy was higher (*p* < 0.05) under RT (−23,274.6 J mol^−1^) than under NT (−1313.73 J mol^−1^). Conclusively, it was shown that the topramezone adsorption capacity was higher under NT, and biochar addition increased more pesticide adsorption under NT than under RT.

## 1. Introduction

Once applied, pesticides dissipate in different compartments of the natural environment through volatilization, training to surface water by runoff, vertical transfer through soils [1] photolysis, and absorption by living organisms. At ground level, two major processes condition the fate of pesticides: degradation (biotic and abiotic) and retention by the solid soil matrix (phenomena of adsorption-desorption). A fraction of the pesticide can remain mobile in the soil solution and constitutes the so-called available fraction. In fact, the pesticide will be available for living organisms (plants, microorganisms), in this case, it is called bioavailability but also for deep entrainment to groundwater, thereby generating their contamination [2]. The retention of pesticides in soils is an essential process because it regulates their persistence, bioavailability, and transfer to surface and underground waters. Topramezone; (3-(4,5-dihydro-1,2-oxazol-3-yl)-4-mesyl-o-tolyl) (5-hydroxy-1-methylpyrazol-4-yl) methanone; is a selective, systemic herbicide that shows effective herbicidal activity in controlling against broadleaf weeds and grasses as well as several aquatic plant species. Topramezone has been shown to be useful as a resistance management tool for growers experiencing target species resistance and tolerance to triazine herbicide and acetolactate synthase (ALS)-inhibitor herbicides [3]. It can be somewhat persistent in aerobic soils. Its overuse can result in serious environmental and health risks. Aerial drift and surface water runoff were identified as potential routes of exposure to topramezone residues in aquatic ecosystems and for non-target terrestrial plants. Some topramezone residues can also be available in irrigation water and can be harmful to irrigated non- target crops. In general, the retention of pesticides at ground level limits their degradation and reduces their leaching to groundwater [4,5]. The adsorption of pesticides by the soil is the process of retention most studied and most known. Sensu stricto adsorption is defined as an interfacial phenomenon that corresponds to the transfer of ions or molecules (pesticides) from a fluid phase (the soil-solution) and their accumulation on the solid phase of the soil composed of minerals and organic matter [6].

Some studies have shown that soil properties and adsorption were enhanced by biochar addition [7]. Biochar, i.e., pyrogenic carbon (C), is made from biomass through the pyrolysis process at 250–800 °C and in oxygen-limited conditions. Biochar porosity will be beneficial to crops to regulate their water consumption according to their needs. Some studies showed that biochar played an important role in enhancing the pesticide adsorption capacity onto loess soil in north-western China [8,9]. Around 35% of Chinese maize production is from the North China Plain [10]. There may be some more pollutants like toxic metals with topramezone; therefore, there would be competitive adsorption, which would probably affect the topramezone adsorption. The toxic metal adsorption should be different from the pesticide adsorption onto soils due to their various chemical properties. Most of the technologically modified adsorbents have an adequate adsorptive capacity [11,12] but are not economically affordable. Therefore, the vast and free waste of post-harvest maize straw has to be treated and might be used for biomass production. So, a study on adsorption behavior of topramezone on soils under tillage management affected by maize straw biochar is needed. In the North China Plain, most of the agricultural activities are done by tillage treatments; therefore, a deepened research on tillage effects with (out) biochar on topramezone adsorption is needed.

In modern agriculture, tillage practices have been extensively used to improve crop quality and production. These agricultural practices are likely to influence the structural properties of the soil, therefore, by the transport of pesticides. The technique of conventional tillage reduces the soil macroporosity and, therefore, limits the transport of phytosanitary products by preferential flow [13]. There is a comparison with conventional tillage and an increase in atrazine leaching in the no-tillage treatments [14]. Many studies suggest that no-tilled soils promote the formation of macropores via the roots of plants and, hence, the risk of transfer through these preferential paths [15]. It was shown, by contrast, that no-till and mulching are beneficial techniques, which, by generating the accumulation of organic matter in the soil, increase the retention of pesticides and, therefore, limit their transfer to the water table [16]. Because of previous works, it is evident that until today, considering the complexity of soil constituents, the impact of different tillage techniques on the transfer and fate of pesticides is not entirely clarified. It would be worth examining the effect of straw maize biochar on topramezone adsorption behavior on soil under no-tillage and rotary tillage treatments since the mechanism of no-tillage and tillage treatments to adsorb pesticides via kinetic and thermodynamic processes is not well known. The aims of this work are to evaluate:

1. Topramezone adsorption behavior under no-tillage and rotary tillage treatments,
2. Biochar addition impact on topramezone adsorption under no-tillage and rotary tillage treatments. It is planned to carry out both a laboratory and field-based study because there may be some differences between the lab analysis results and what really happens in the natural cropping system, which should be further examined in detail.

## 2. Materials and Methods

### 2.1. Chemicals

Topramezone standard of high purity (99.4%) was purchased from Hangzhou Dayangchem limited company. Topramezone stock solution was prepared in acetonitrile, and other manipulating solutions (such as calcium chloride) were prepared by adding de-ionized water to the stock solution.

#### 2.1.1. Maize Straw and Soil Sampling 

The sampling area for both soil and biochar feedstock was selected in Luancheng Agro-Ecosystem Experimental Station, in Hebei Province, Northern China. The feedstock was maize straw, which was divided into 2 cm pieces and dried at 70 °C in an oven.

Samples were collected from 20 cm soil depth with a soil sampler (probe stainless steel T-style soil test kit) for both no-tillage and rotary tillage treatments (5 m of between space), then air-dried, sieved through a 2 mm sieve, and stocked in respective plastic bags at room temperature. The standard methods were used to determine the soil physico-chemical properties [17,18]: the pH of soil was determined in a 1:5 soil to water suspension using a pH meter (METTLER TOLEDO, Instrument Co.Ltd, Shanghai, China), the electrical conductivity was measured by a conductivity meter SG7 with a glass electrode (METTLER TOLLEDO, Instrument Co.Ltd, Shanghai, China), the soil organic matter determined by Walkley-Black method and soil particle structure analysis carried out using a Mastersizer 3000 laser diffractometer, equipped with Hydro-Lv dispersion unit (Hydro-Lv MAZ 3300-Mastersizer 3000 Hydro-series Malvern, Malvern Panalytical SARL, Orsay Cedex, France).

#### 2.1.2. Biochar Preparation

The dried biomass samples were placed in crucibles, covered with lid and exposed to N_2_ gas with the flow rate of 4.16 L min^−1^ pyrolyzed under limited oxygen-heating conditions in a Microwave Muffle Furnace (Phoenix type CEM Shanghai, China) then heated to 300, 400, and 500 °C with increasing rate of 5 °C min^−1^. The maximum temperatures were constant for 1 h, then the samples were cooled to 100 °C, and after turning off the N_2_ gas exposure, the carbonization products corresponding to the respective temperature were obtained. Samples were labeled as MBC-300 (maize biochar prepared at 300 °C), MBC-400 (maize biochar prepared at 400 °C), and MBC-500 (maize biochar prepared at 500 °C). Maize straw biochar samples were sieved to <0.2 mm, and their physico-chemical properties were examined.

### 2.2. Biochar Characterization

Maize Biochar samples (MBC300, MBC400, and MBC500) were characterized as follows: The elemental C and N concentrations were determined with an elemental analyzer (vario PYRO cube, Elementar, Germany). The BET (Brunauer–Emmett–Teller) surface area and pore volume of maize straw biochars were determined from adsorption isotherms using a Porosity Analyzer (V-Sorb 2800P, Beijing, China). Scanning electron microscopy (SEM, ZEISS Gemini FESEM) for biochar morphology characterization and Fourier Transform Infra-Red Spectrometry (FTIR, Nexus 670, NIST, Gaithersburg, USA) for biochar chemical functional groups characterization using KBr pellets to run 20 scans per run samples were carried out.

### 2.3. Batch Adsorption Experiments

#### 2.3.1. Adsorption Kinetics

The adsorption kinetics of the topramezone on soil with or without biochar were tested using a batch equilibrium method, as described previously [19,20,21]. The adsorption kinetics were determined at a constant temperature (25 ± 0.2 °C), incubating 0.5 g of soil with 0.02 g of biochar (particle size at 0.2 mm), and 10 mg L^−1^ of topramezone (V = 1 mL) diluted in a 0.01 M CaCl_2_ solution at different time intervals (0, 1, 2, 4, 6, 8, 12, 16, 20, 24 h) in 50 mL PVC tubes to prevent from the dissolved organic matter leaching during the adsorption process, under shaking at 200 rpm, and samples were analyzed for residual topramezone concentration using UV-vis spectrophotometer (UV-2450 SHIMADZU, Tokyo, JAPAN) at 256 nm wavelength.

#### 2.3.2. Adsorption Thermodynamics

For the thermodynamic adsorption study, 0.5 g of soil and 0.02 g of biochar were mixed with different topramezone concentrations ranging from 0 to 16 mg L^−1^, in 50 mL PVC tubes at constant agitation speed of 200 rpm for 12 h under different temperatures (15, 25, and 40 °C) shaking, after 2 h samples were centrifuged at 4000 rpm for 15 min and analyzed using UV-vis spectrophotometer as referenced in the kinetic experiments section at the same wavelength.

The adsorption capacity (q_s_, mg g^−1^) was calculated from the difference between the initial and equilibrium topramezone concentrations according to the following equation:

(1) $$q_{s}=\frac{\left(C_{0}-C_{e}\right)\times V}{m}$$

with C_o_: the initial topramezone concentration (mg L^−1^), C_e_: equilibrium pesticide concentration, i.e., final (mg L^−1^), V: liquid phase volume (mL) and m: adsorbent mass (mg).

#### 2.3.3. Thermodynamic and Kinetic Modeling

Thermodynamics 

Langmuir, Freundlich, and Dubinin–Radushkevich (DR) isotherm models were used to assess the Imidacloprid adsorption on the soil.

(2) $$\frac{1}{q_{s}}=\frac{1}{K_{L}Q_{m}C_{e}}+\frac{1}{Q_{m}}$$

(3) $$\mathrm{lg}q_{s}=\mathrm{lg}K_{F}+\frac{1}{n}\mathrm{lg}C_{e}$$

(4) $$\ln q_{s}=\ln Q_{m}-\beta \varepsilon^{2}$$

The linear Langmuir, Freundlich, and DR equations are shown in Formulas (2)–(4) respectively, with, C_e_: the equilibrium concentration of topramezone in the liquid phase, mgL^−1^, q_s:_ the adsorption capacity of Imidacloprid in the soil samples, mg g^−1^, Q_m_: Topramezone adsorption capacity, mg g^−1^; K, n and b are constants associated with soil properties [22,23,24].

The model was used to differentiate the physical adsorption from the chemical one. With its mean free energy, E per molecule of adsorbate (for removing a molecule from its location in the sorption zone to the infinity) can be obtained by the equation:

(5) $$E\;=\;\frac{1}{\sqrt{2(-B)}}.$$

Adsorption data at different temperatures are plotted as a function of the logarithm of amount adsorbed versus the square of potential energy:

Ε = RTln(1 + 1/C_e_). (6)

Many works calculated the Gibbs free energy using Kc as a constant with units, which was not adequate.

Equations (8) and (9) are used to calculate the adsorption Gibbs free energy ΔG, change in enthalpy (ΔH), and change in entropy (ΔS)

K_c_ = 55.5K_L_, (7)

ΔG = −RTlnK_c,_ (8)

lnK_c_ = ΔH/RT + Δ/R, (9)

with R: molar constant of the ideal gases; K_c_: dimensionless adsorption equilibrium constant [25]; T: operating system temperature. From the lnKc~1/T plot, according to the straight-line slope and intercept, enthalpy (ΔH) and entropy changes (ΔS) are calculated, respectively. Adsorption isotherms are important data in the adsorption mechanism understanding.

##### Kinetics

The kinetic study is important to an adsorption process because it underlines the uptake rate of adsorbate and controls the residual time of the whole process. Three kinetic models, pseudo first-order, pseudo second-order, and intra-particle diffusion, were used in this work for describing the adsorption process.

The three models represented, respectively, as linear Equations (5)–(7) are the following:

(10) $$\frac{1}{q_{t}}=\frac{1}{q_{1}}+\frac{k_{1}}{q_{1}\times t},$$

(11) $$\frac{t}{q_{t}}=\frac{1}{k_{2}\times q_{2}^{2}}+\frac{t}{q_{2}},$$

(12) $$q_{t}=k_{p}\times t^{1/2}+C,$$

where: t: adsorption time in min; q_1_ and q_2_: for the equilibrium adsorption capacity mg/g; q_t;_ the adsorption capacity mg g^−1^; when k_1_ and k_2_, respectively, are pseudo first-order kinetic rate constants of adsorption, and C is the intercept.

### 2.4. Statistical Analysis

In order to analyze and design the adsorption process, different adsorption isotherm, kinetic models, and thermodynamic equations were applied to fit the experimental data to find out appropriate models to predict isotherm, kinetic, and thermodynamic data.

## 3. Results and Discussion

### 3.1. Biochar Characterization

**Figure 1:**
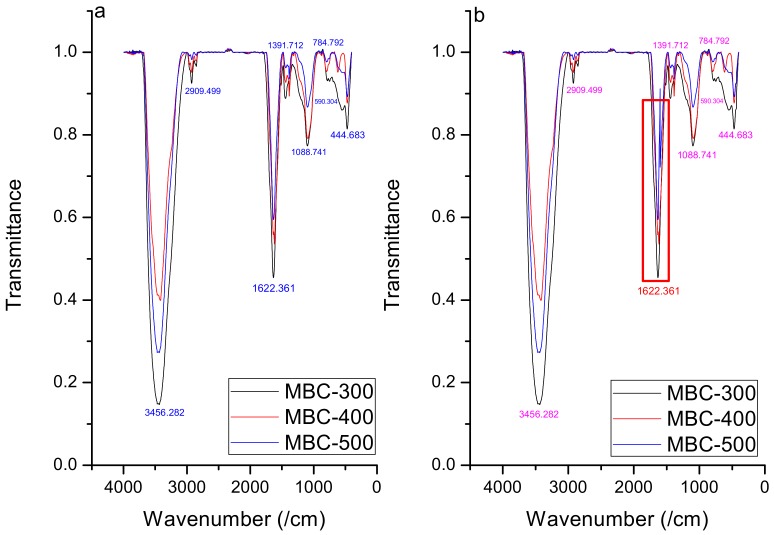
Fourier Transform Infra-Red Spectrometry (FTIR) spectra of biochar samples (**a**) before and (**b**) after topramezone adsorption (the unity corresponds to 100 percent of transmittance).

The maize straw biochar chemical functional groups were illustrated by FTIR (Figure 1) at respective pyrolytic temperatures. 

The spectra of biochar were characterized at wave numbers 3431, 2909, 2302, 1622/1412, 1391/1225, 1088/1051, 784/735, 590, and 444 cm^−1^, which correspond to the stretching of hydroxyl (–OH), methylene (–CH_2_–), carbon-carbon or nitrogen-nitrogen triple bond, aromatic carboxyl/carbonyl (–C=O), –COOH and –CHO, aromatic CO– and phenolic –OH, C–X C–O–C, respectively [26]. The intensity was higher in MBC-300 compared to MBC-400 and MBC-500. FT-IR analysis of three biochars indicated broad peaks between 3456 and 3431 cm^−1^ (Figure 1) corresponding to O–H stretching for alcohols and phenols [27], and it was weak in MBC-500 and MBC-400, indicating water loss with increasing temperature. Significant bands also occurred between 2909 and 2823 cm^−1^, which refer to the vibrations of strong C–H bond in aldehydes methyl, or alkanes, and the bands dwindled or disappeared at higher pyrolytic temperature (500 °C), indicating the abundance of aliphatic compounds. FTIR peaks between 1622 and 1412 cm^−1^, which correspond to esters and aromatic C=C or C=O stretch in carboxylates [28], appeared and may involve both basic and acidic groups, which may probably result from a basicity increase with corresponding pyrolytic temperatures. Most of these bands also weakened at 500 °C as C=O ruptures to form liquids and gases, while esters revoke the development of lactones [29]. Peaks appeared at 1391/1225 cm^−1^ indicated the presence of –COOH and –CHO; the acidic functional groups decreased with increasing temperature, and the acidic groups were nearly absent at higher temperatures indicating that cellulose was decomposed and C–O–C was broken and lignin was decarboxylated [30]. The other significant bands at 1095, 787, and 509 cm^−1^ for aromatic C–H bend and a regular C–X stretching of halides or strong C–H stretches of tri-substituted alkenes C–N stretch of aliphatic amines, respectively, also may be due to the inorganic mineral composition of the biochar [31]. The change in absorption band at 1622 cm^−1^ after adsorption in MBC-500 may result from the C=O breaking caused by both high pyrolytic and system temperatures.

As the surface area is an essential property for adsorbing substances, the surface area of biochar was, therefore, illustrated to analyze adsorption performance. The results (Table 1) based on calculations of standard BET equation (2.063, 20.208, and 20.89, for MBC-300, MBC-400, and MB-C500, respectively), Langmuir (2.978, 30.339 and 40.556, for MBC-300, MBC-400, and MB-C500, respectively), and BJH (Barret-Joyner-Halenda) adsorption (2.191, 21.780, and 30.295, for MBC-300, MBC-400, and MB-C500, respectively) showed that the surface area on biochar increased with increasing pyrolytic temperature, this may be due to the removal of mobile matter.

**Figure 2:**
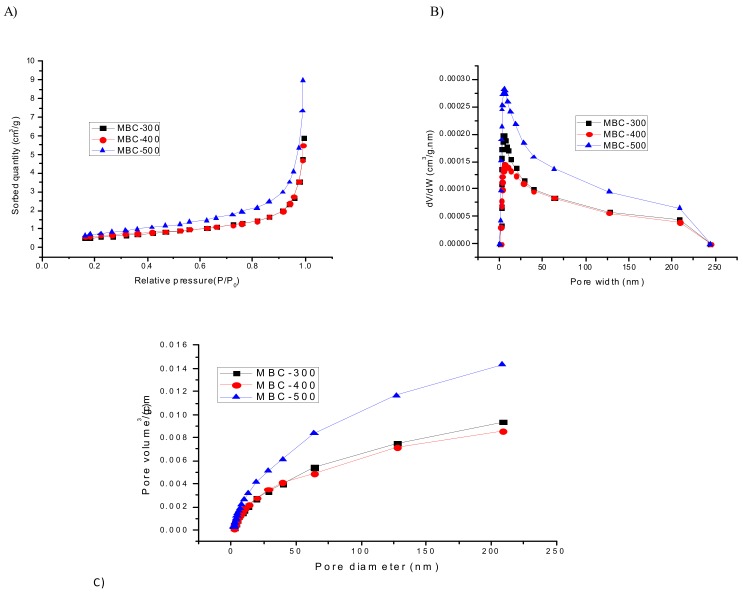
(**A**) N_2_ adsorption-desorption isotherm curves of Maize straw biochars, (**B**) BJH (Barret-Joyner-Halenda) pore size distribution curves of MBC-300, MBC-400, and MBC-500, and (**C**) Maize straw biochars BJH-adsorption-pore size distribution.

However, a large surface area of maize biochar does not mean that the biochar possesses a higher adsorption capacity. Because surface area may not be the only factor for adsorption on MBC, topramezone adsorption could probably depend on the surface chemistry. As shown in Figure 2A, the adsorption curve of MBCs was type III, which indicates a physical adsorption process on the macroporous adsorbent. Figure 2A shows that the adsorption capacity increased very fast at high pressure (P/P0 > 0.9), while the adsorption rate increased with lower pressure (P/P0 < 0.2), which indicates a large number of microporous structures [32].

The pore structure can also be explained from the BJH pore size distribution curve of MBCs (Figure 2B).

**Figure 3:**
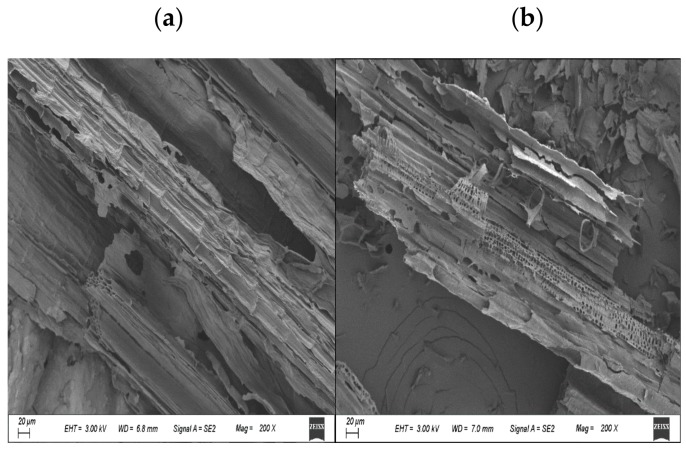
Scanning electron microscopy (SEM) images of the MBC-400 sample: (**a**) before and (**b**) after topramezone adsorption.

Previous studies concluded that the biochars produced at <450 °C had low porosity due to the covering of pores by volatile organic compounds, thus could affect adsorption capacity [33]. In this study, MBC-500 was found more porous than MBC-300 and MBC-400 for small pore size (<45 nm) (Figure 2C and Figure 3), which resulted in an increased surface area [34], and with a larger pore diameter, the MBC adsorption capacity is reduced, which this may be due to high pyrolytic temperature resulted in a complete destruction of the original structures.

### 3.2. Batch Experiments

#### 3.2.1. Adsorption Thermodynamics

**Figure 4:**
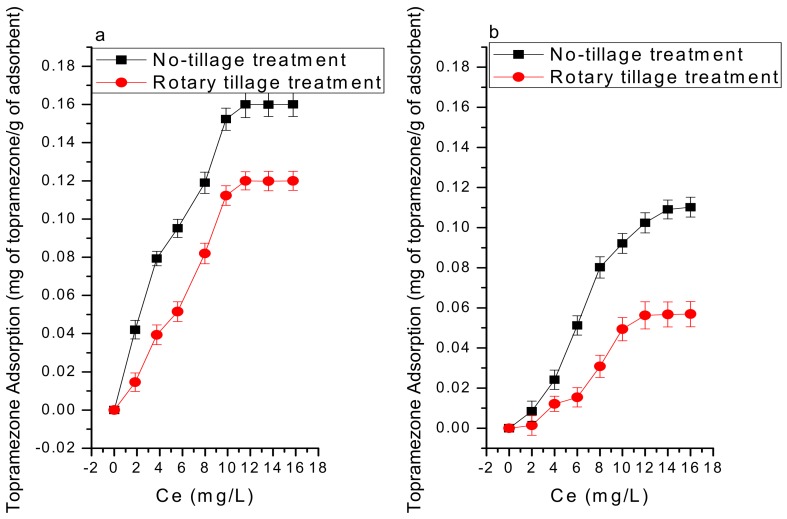
Adsorption of Topramezone under no-tillage and rotary tillage treatments (**a**) in the presence of maize straw biochar produced at 400 °C, (**b**) without biochar.

The effect of temperature on adsorption was significant. This increase can be attributed to the increased surface coverage of topramezone at higher temperatures due to the expansion of new active sites on MBC. The large size of topramezone and the presence of inorganic metals (shown in Table S1) in MBC could be the reason for slightly better adsorption at higher operating temperatures. Topramezone is a polar substance (logKow = −1.52 at 20 °C, pKa = 4.6 at 25 °C) and retained more on no-tilled soil than on rotary tilled soil, which may be of higher clay content, organic matter, and cation exchange capacity (Figure 4). This adsorption mechanism could also be explained by H-bondings between atoms of topramezone and adsorbents.

The difference in electrical conductivity in no-tillage and rotary tillage treatments (Table 2) may result from mechanical tillage activity that could render more salts available in the soil after fertilization and irrigation activities. The electron movement in the soil profile is a complex mechanism. The exchangeable ions of soil minerals, the soil electrical conductivity depends on different factors, such as density, organic matter, and electrolytes, structure, and the mineral phase conductivity, which can affect topramezone adsorption.

Evaluating the soil electrical conductivity with the effect of such factors is an area where little research has been done. There must be more research about the change in electrical conductivity impact on pesticide adsorption onto a soil profile. The increase in adsorption with the topramezone concentration could be explained by the fact that during the process, the boundary layer film onto the adsorbent surface is diffused by the topramezone molecules migrating finally into the porous structure of the biochar [35,36]. The electrical conductivity was found to be higher in rotary tillage than in no-tillage treatment; 162.2 and 115.1 µS/cm, respectively. This significant difference may also be because the soil humidity is maintained and infiltrated in deep layers of no-tillage treatment, whereas the significant part of tillage treatment, water evaporates into the atmosphere resulting in the accumulation of water ionic salinity onto the soil surface [37]. It may also result from the difference in evapotranspiration and infiltration. The high adsorption increase under no-tillage could also be attributed to the higher total organic and clay contents than under rotary tillage treatment.

The adsorption capacity (Qm) value shows the total adsorption capacity of soil particle for topramezone, and it was affected by biochar addition. The Qm value decreased with increasing temperature for both treatments, but the Qm was greater under no-tillage than the rotary tillage treatment (Table 3). This could be explained by the porous structure of biochar that retains topramezone molecules. Qm decreased with increasing temperature (15 > 25 > 40 °C), suggesting an exothermic process; 0.217–0.088 mg g^−1^ and 0.143–0.073 mg g^−1^, for no-tillage and rotary tillage treatments, respectively (Table 3).

The topramezone adsorption and the Langmuir model matched well, where R^2^ values were also higher for no-tillage (0.903 to 0.989) than rotary tillage’s treatment (0.892 to 0.978). The isotherm obtained follows the Langmuir equation with a good approximation (R^2^ = 0.95), which indicates that the adsorption was fundamentally governed by topramezone partition between the soil, soil-water mixture, and a monolayer adsorption. In this study, ΔH ranges from −1.313 to −64.670 kJ mol^−1^ (Table 3), which indicates that the adsorption is an exothermic process. The Qm increases with an increase in the pyrolytic biochar temperature, as shown in the no-tillage treatment (from 0.088 to 0.217 mg g^−1^) and for the rotary tillage treatment (0.073 to 0.143 mg g^−1^), probably due to the porous structure of the biochar. Qm also significantly decreased with temperature after MBC-400 addition, 15 > 25 > 40 °C, for no-tillage treatment (from 0.210 to 0.129 mg g^−1^) and for rotary tillage treatment (from 0.143 to 0.094 mg g^−1^) (Table 3), which is probably because topramezone is a non-ionic organic molecule and it is, therefore, retained by a physical adsorption process. It may also result that from lower temperatures, organic molecules tend to precipitate and, hence, are readily adsorbed. The solute competes with the solvent for the occupation of fixation sites present on the solid phase. These curves are characteristic of monofunctional organic compounds with moderate intermolecular attractions. The addition of MBC-300 decreased the adsorption of topramezone, probably because the maize straw was not carbonized at a low temperature. The regression coefficients (r^2^) were the highest for the Langmuir model compared to two other models, implying that the isothermal adsorptions of topramezone under both treatments were well described by the Langmuir model.

The adsorption capacity increases with the increase in pyrolytic temperature, with the exception where MBC500 ≤ MBC400 and MBC500 < MBC400, for no-tillage and rotary tillage treatments, respectively, which may be due to the total carbonization and the high mineral content of the maize biochar produced at 500 °C.

SEM shows that temperature was an influential factor that affects the surface properties of maize straw derived biochar (Figure S1). The surface properties were changed with increasing temperatures. At 300 °C, the pore channels of straw were regularly dispatched, while at 400 °C, pore channels were carbonized into pieces, and it was more evident at 500 °C.

**Figure 5:**
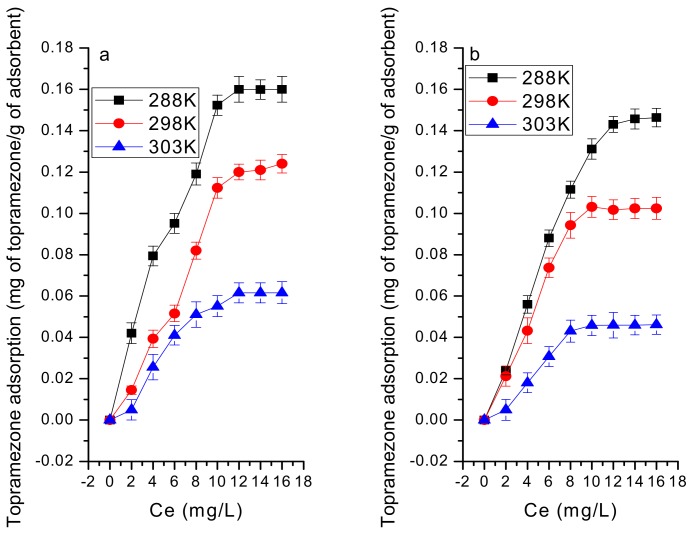
Sorption isotherms of Topramezone under (**a**) no-tillage treatment and (**b**) rotary tillage treatment affected by MBC-400 under different system temperatures.

A previous study also found a similar decrease in the pore channels in biochar with increasing pyrolytic temperature [38]. As temperature increases (exceed to 400 °C), the higher released energy can cause melting and disorganization of pore walls and, thus, can improve the biochar surface roughness. The topramezone adsorption has been found more exothermic under rotary tillage than no-tillage treatment (Table 4). This may be due to the higher electrical conductivity, resulting in higher soil mineralization in the rotary tillage treatment, which should release more heat during the adsorption process. This has been reconfirmed by the thermodynamic parameters where the rotary tillage treatment ΔH is 17 times higher than no-tillage treatment’s ΔH, −23,274.6 and −1313.73 J mol^−1^, respectively. The adsorption was higher at 15 °C than at 25 and 40 °C for both tillage treatments; this may result from its high electrical conductivity and soil temperature (Figure 5). 

This significant difference (Table 3) in the negative values for the change in enthalpy (ΔH) for no-tillage and rotary tillage treatments may result from the fact that the rotary tilled soil is dryer than the no-tilled one [39], since the electrical conductivity is proportional to the temperature [40].

Additionally, this could also be explained by the negative values of the change in enthalpy (ΔH) shown in the thermodynamic parameters, from −46,169.5 to −1313.73 J mol^−1^ and from −102,196 to −23,274.6 J mol^−1^, a significant difference, for no-tillage and rotary tillage treatments, respectively. The negative value of ΔS (change in entropy) show a decreased randomness at the adsorbent and solution interface, i.e., an increase in the order of the system. The negative values of ΔG (Gibb’s free energy) for the biochars indicate the spontaneity of the adsorption process. Moreover, during the process, cracking and volume shrinkage would change the soil texture. Thus, the tensile strength is further reduced, and more cracks are created. The change in the inner soil structure could affect the electrical conductivity and decrease residual forces on the soil surface, which results in a decreased soil surface energy. The negative value of ΔS (change in entropy) indicates a decreased randomness during adsorption, which, at higher temperatures, contributed to the decrease in topramezone adsorption capacity [41]. The negative values of ΔG (Gibb’s free energy) showed that the adsorption process was spontaneous, as indicated in Table 4.

#### 3.2.2. Adsorption Kinetics

**Figure 6:**
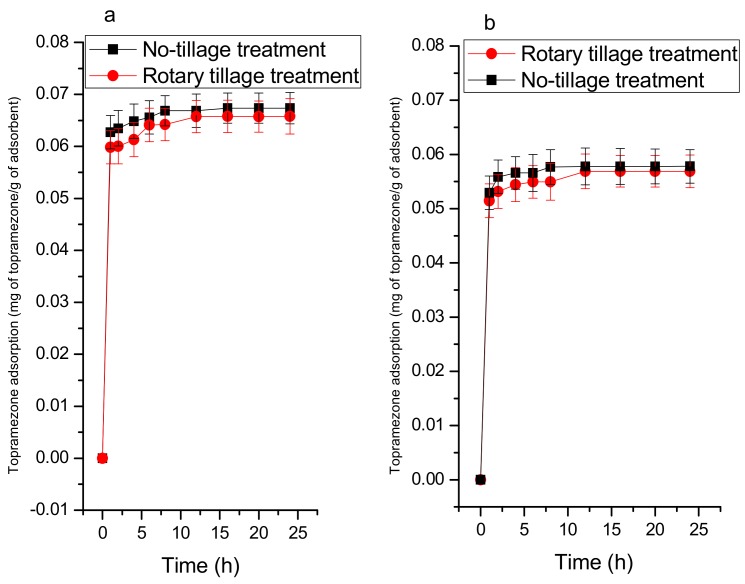
Kinetic adsorption of Topramezone under no-tillage and rotary tillage treatments in (**a**) the presence and (**b**) absence of maize straw biochar (produced at 400 °C).

Topramezone was adsorbed quickly during the first stage because the vacant sites were still available, followed by slow diffusion of topramezone molecules into the soil. This was also found by [42]. There is an effect of time on the amount of topramezone adsorption on soil with added biochar. Before and after adding MBC-400 (Figure 6), the equilibrium reached after 12 and 8 h for rotary tillage and no-tillage treatment, respectively. The topramezone adsorption was found higher under no-tillage than rotary tillage treatment (Figure 6), this may result from the clay content, and organic matter in the no-tillage treatment, and it may also be due to the high mineral content and high electrical conductivity in the rotary tillage treatment. After adding biochar, the topramezone was more adsorbed than before biochar addition, and this may result from biochar having more vacant sites to occupy topramezone molecules. The kinetics could also be affected by the pores blocking due to the high molecular mass of the adsorbate.

The kinetic data show that the adsorption of topramezone onto both soils could be described by a pseudo-second-order kinetic model, where some R^2^ values are higher than 0.9 (Table 5). Also, it is of note that the experimental q_t_ and calculated q_2_ values are in a better agreement for a pseudo-second-order kinetic model than the pseudo-first-order kinetic model [43]. The pseudo-first-model R^2^ values and kinetic constants are too low, and this may be due to the fact that the influence of the temperature is small on the kinetic constant whatever the porous structure of the adsorbent particle, which suggests that this adsorption is not a pseudo-first-order reaction [44,45].

As a recommendation, highly modified adsorbents should be used to immobilize and degrade pollutants (pesticides, dyes, etc.) in the environment [46].

#### Effect of pH on Topramezone Adsorption

**Figure 7:**
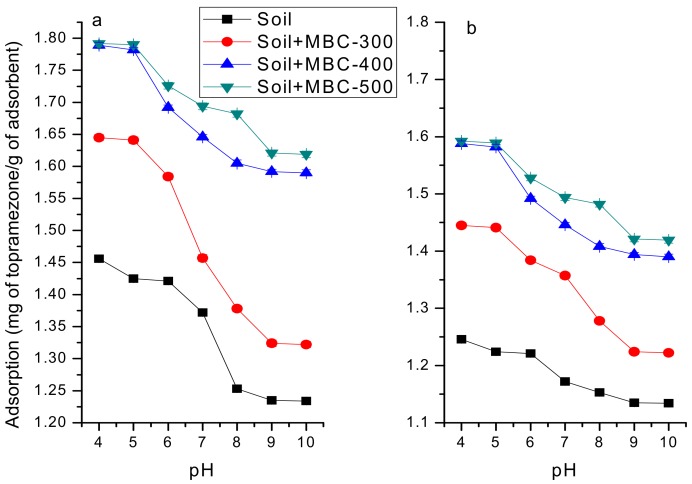
Effect of pH on topramezone adsorption on (**a**) soil under no-tillage treatment and (**b**) rotary tillage treatment affected by biochar.

pH may affect the adsorption of organic compounds onto biochar in polluted aqueous solutions. It is also an important factor that has an effect on the fate of organic pollutants in soil. Hereby, it is necessary to examine how pH affects the adsorption of topramezone on soil under no-tillage and rotary tillage treatments with added biochar (Figure 7). The adsorption was greater under NT than under RT.

The adsorption capacity was weakened as pH increased. With the increase in pH less than 7, the adsorption capacity decreased slowly, and decreased rapidly with the increase in pH > 7, suggesting that the adsorption of topramezone on soil under no-tillage and rotary tillage treatment was favored under acidic conditions. The decrease was greater under no-tillage treatment than under rotary tillage treatment. This is probably because pH was affected not only by the colloid charge distribution of the soil but also by the distribution of various topramezone components.

## 4. Conclusions

The biochar produced from maize straw residue had more effect on topramezone adsorption under no-tillage than under rotary tillage treatment. The adsorption kinetics and thermodynamics matched well with the pseudo-second-order kinetics equation and the Langmuir isotherm models, respectively, indicating a monolayer layer adsorption of topramezone onto biochar. The adsorption capacity was enhanced with the decrease of the system temperature, indicating that the adsorption was an exothermic process. The negative ΔG values suggest the spontaneous adsorption of pesticide on biochar. The rotary tillage treatment’s ΔH value was significantly higher than no-tillage treatment, which may result from the individual differences in soil temperatures and electrical conductivity values; therefore, more experimental works are necessary for the future. Further research should focus on the pesticides fate and transport, specifically emphasizing soil properties changed by tillage treatment and enhancing possible ways for their retention by sorbents, such as biochar, as it has been stated in this study.

## Figures and Tables

**Table 1:** Biochar specific surface area and pore characteristics.

Biochar Sample	BET Surface Area (m^2^/g)	BJH Adsorption Cumulative Surface Area (m^2^/g)	Langmuir Surface Area (m^2^/g)	Pore Diameter (nm)	BJH Adsorption Average Pore Diameter (nm)	SF Micro Pore Volume cm^3^/g
MBC-300	2.063	2.191	2.978	17.749	17.089	6.16 × 10^−4^
MBC-400	20.286	21.780	30.339	14.822	18.820	7.86 × 10^−3^
MBC-500	20.897	30.295	40.556	19.208	17.424	8.99 × 10^−3^

**Table 2:** Soil physicochemical properties.

Parameter	Rotary Tillage Treatment	No-Tillage Treatment
	Value	Value
Sand (%)	24.07 ± 0.04	21.64 ± 0.02
Silt (%)	60.02 ± 0.02	60.12 ± 0.02
Clay (%)	15.91 ± 0.02	18.24 ± 0.01
pH	8.37 ± 0.04	8.36 ± 0.04
Electrical Conductivity (µS/cm)	162.20 ± 0.01	115.10 ± 0.01
Cation Exchange Capacity (meq/100 g)	14.03 ±0.02	15.11 ± 0.00
Total Organic Carbon (%)	1.583 ± 0.00	1.734 ± 0.00
Total Organic Nitrogen (%)	0.148 ± 0.00	0.158 ± 0.00
Bulk Density	1.40 ± 0.02	1.43 ± 0.02

**Table 3:** Eigen value of isothermal adsorption equation of Topramezone under no-tillage and rotary tillage treatments affected by biochar.

			Langmuir Equation			Freundlich Equation			D-R Equation		
		K_L_(L/mol)	Q_m_ (mg/g)	r^2^	K_F_((mol/g) (L/mol)^1/n^)	n	r^2^	Β (mol^2^/J^−2^)	lnQ_m_ (mol/g)	E (J/mol)	r^2^
**15 °C**	***Soil NT***	0.039	0.129	0.926	0.005	0.759	0.807	−5.4×10^−8^	−0.142	9512.02	0.739
	***RT***	0.021	0.065	0.914	0.0039	0.762	0.801	−5.6 ×10^−8^	−0.124	9482.02	0.714
	***S+MBC300 NT***	0.163	0.187	0.972	0.015	1.092	0.865	−2.3 ×10^−8^	−0.225	14,821.14	0.554
	***RT***	0.125	0.098	0.968	0.015	1.094	0.845	−2.4 ×10^−8^	−0.208	14,515.12	0.558
	***S+MBC400 NT***	0.225	0.21	0.989	0.047	1.927	0.927	−1.7 ×10^−9^	−0.252	60,271.07	0.625
	***RT***	0.218	0.143	0.978	0.042	2.001	0.889	−1.9 ×10^−9^	−0.247	60,249.52	0.611
	***S+MBC500 NT***	0.217	0.217	0.984	0.046	1.873	0.974	−2.2 ×10^−9^	−0.250	49,020.00	0.646
	***RT***	0.202	0.146	0.972	0.042	1.881	0.965	−2.2 ×10^−9^	−0.241	49,020.00	0.651
	***Soil NT***	0.04	0.085	0.914	0.0006	0.559	0.83	−5.2 ×10^−8^	−0.215	9456	0.762
	***RT***	0.028	0.059	0.902	0.0006	0.584	0.825	−5.3 ×10^−8^	−0.210	9446.019	0.658
**25 °C**	***S+MBC300 NT***	0.125	0.15	0.94	0.0007	0.536	0.903	−2.0 ×10^−8^	−0.279	13,074.14	0.656
	***RT***	0.012	0.121	0.923	0.0005	0.561	0.901	−2.1 ×10^−8^	−0.244	49,520.00	0.627
	***S+MBC400 NT***	0.158	0.178	0.962	0.0081	0.944	0.927	−1.3 ×10^−9^	−0.282	57,071.02	0.62
	***RT***	0.032	0.123	0.928	0.0062	1.021	0.901	−1.3 ×10^−9^	−0.277	57,071.02	0.614
	***S+MBC500 NT***	0.165	0.175	0.961	0.0157	1.247	0.926	−2.1 ×10^−8^	−0.272	49,520.00	0.642
	***RT***	0.033	0.122	0.954	0.0095	1.243	0.913	−2.3 ×10^−8^	−0.213	14,821.14	0.645
	***Soil NT***	0.04	0.085	0.903	0.00005	0.556	0.86	−5.4 ×10^−8^	−0.174	9512.02	0.79
	***RT***	0.023	0.048	0.892	0.00005	0.562	0.885	−5.4 ×10^−8^	−0.145	9512.02	0.741
**40 °C**	***S+MBC300 NT***	0.036	0.088	0.938	0.00017	0.178	0.88	−2.3 ×10^−8^	−0.225	14,821.14	0.589
	***RT***	0.011	0.073	0.92	0.00011	0.183	0.819	−2.6 ×10^−8^	−0.158	13,846.02	0.582
	***S+MBC400 NT***	0.08	0.129	0.938	0.0026	0.478	0.927	−2.1 ×10^−8^	−0.225	49,520.00	0.589
	***RT***	0.03	0.094	0.933	0.0027	0.478	0.921	−2.4 ×10^−8^	−0.166	14,515.12	0.603
	***S+MBC500 NT***	0.079	0.134	0.935	0.0048	0.402	0.804	−2.3 ×10^−8^	−0.164	14,821.14	0.578
	***RT***	0.031	0.098	0.933	0.0041	0.411	0.905	−2.3 ×10^−8^	−0.152	14,821.14	0.57

**Table 4:** Thermodynamic parameters for the topramezone sorption under no-tillage and rotary tillage treatments affected by biochar.

**SOIL**	**T**	**ΔG**	**ΔH**	**ΔS**
	**(K)**	**(J/mol)**	**(J/mol)**	**(J/MOL/K)**
	288	−1848.07 −366.531		
	298	−1974.93 −1091.67	−1313.73 −23,274.6	−21.92 −78.296
	303	−2008.07 1242.541		
**MBC300**	288	−5270.91 −4635.64		
	298	−4796.6 1006.563	−64,670.1 −95,149	−134.89 −419.655
	303	−1742.78 1242.541		
**MBC400**	288	−6042.38 −5966.74		
	298	−5376.77 −1422.34	−46,169.5 −10,2196	−193.24 −335.126
	303	−3753.36 −1283.7		
**MBC500**	288	−5955.74 −5784.31		
	298	−5484.12 −1498.54	−44,328.9 −96,562.2	−53.15076 −316.137
	303	−3721.69 −1366.27		

T: Temperature, ΔG: Gibbs free energy, ΔH: change in enthalpy, ΔS: change in entropy. Underlined: No-tillage treatment.

**Table 5:** Eigenvalue for the kinetic sorption equation of topramezone under no-tillage and rotary tillage treatments affected by biochar.

	Pseudo-First Order Equation				Pseudo-Second Order Equation			Intraparticle Diffusion Equation	
	q_1 (mg g_^−1^_)_	k_1(min_^−1^_)_	r_1_^2^	q_2 mg g_^−1^_)_	k_2(gmg−_^1^_min_^−1^_)_	r_2_^2^	k_p (gmg−_^1^_min_^−1/2^_)_	c_p_	r_p_^2^
***Soil NT*** ***RT***	0.089 0.084	0.075 0.070	0.211 0.188	0.021 0.020	22.956 23.574	0.950 0.942	0.003 0.001	0.032 0.0512	0.335 0.328
***Soil+MBC300NT*** ***RT***	0.092 0.090	0.076 0.075	0.208 0.204	0.022 0.021	22.006 22.537	0.950 0.943	0.008 0.005	0.056 0.047	0.735 0.562
***Soil+MBC400NT*** ***RT***	0.103 0.100	0.074 0.074	0.507 0.206	0.025 0.024	19.842 20.294	0.952 0.951	0.012 0.008	0.062 0.060	0.919 0.732
***Soil+MBC500NT*** ***RT***	0.104 0.061	0.076 0.069	0.504 0.304	0.025 0.024	19.648 19.947	0.949 0.937	0.011 0.007	0.063 0.060	0.696 0.654

## Supplementary Materials

The following are available online at https://www.mdpi.com/1660-4601/16/24/5034/s1, Figure S1: Scanning Electron Microscope (SEM) images of A) MBC-300, B) MBC-400 and C) MBC-500 magnified 200 and 1000 times respectively. Table S1: Elemental composition of the straw maize biochars produced at different pyrolytic temperatures title.
