# Supplementary material for: Evaluating Biochar Impact on Topramezone Adsorption Behavior on Soil under No-Tillage and Rotary Tillage Treatments: Isotherms and Kinetics
Source: Int J Environ Res Public Health. 2019 Dec 10;16(24):5034. doi: 10.3390/ijerph16245034 (PMC6950680; doi:10.3390/ijerph16245034)
Supplement: Supplementary file 1 [file ijerph-16-05034-s001.pdf]

## Supplementary

**Table S1** Elemental composition of the straw maize biochars produced at different pyrolytic temperatures

| | **MBC-300** | **MBC-400** | **MBC-500** |
|---|---|---|---|
| **C (g/kg)** | 51.49 | 53.539 | 52.71 |
| **N (g/kg)** | 2.41 | 1.97 | 2.02 |
| **Na(mg/kg)** | 0.33 | 0.42 | 0.61 |
| **Mg(g/kg)** | 5.30 | 4.28 | 7.19 |
| **K(g/kg)** | 19.27 | 32.92 | 38.10 |
| **Ca(g/kg)** | 13.54 | 8.78 | 15.99 |
| **Mn(g/kg)** | 0.30 | 0.32 | 0.32 |
| **Fe(g/kg)** | 3.91 | 2.14 | 7.01 |
| **Cr(mg/kg)** | 3.91 | 9.18 | 5.99 |
| **Ni(mg/kg)** | - | 6.89 | 5.09 |
| **Cu(g/kg)** | 0.12 | 23.80 | 13.03 |
| **Zn(mg/kg)** | 0.33 | 112.89 | 61.95 |
| **Cd(mg/kg)** | - | 0.60 | 0.39 |
| **Pb(mg/kg)** | - | 8.89 | 7.45 |

A 200 times magnified 1000 times magnified

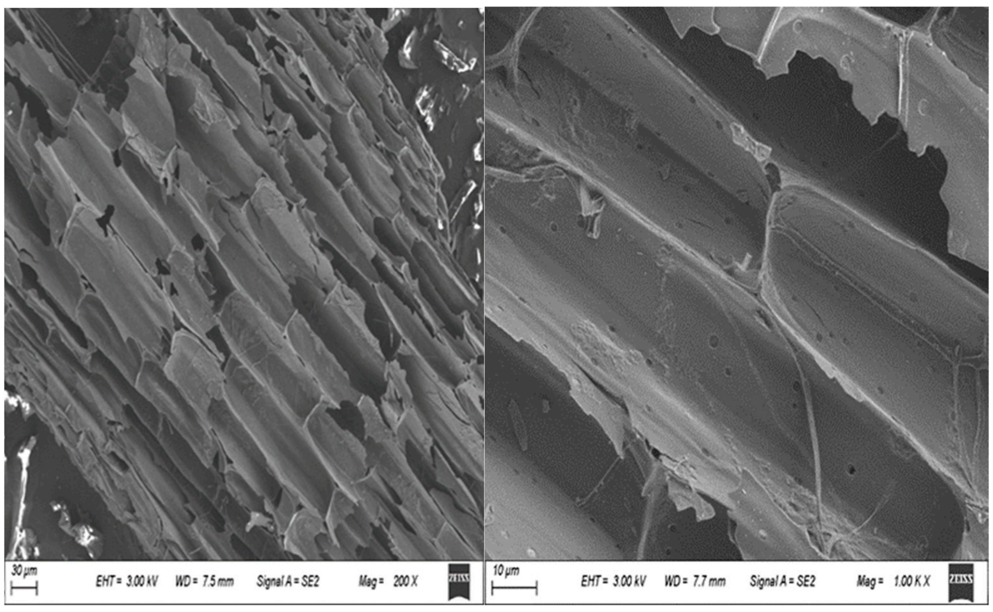


B

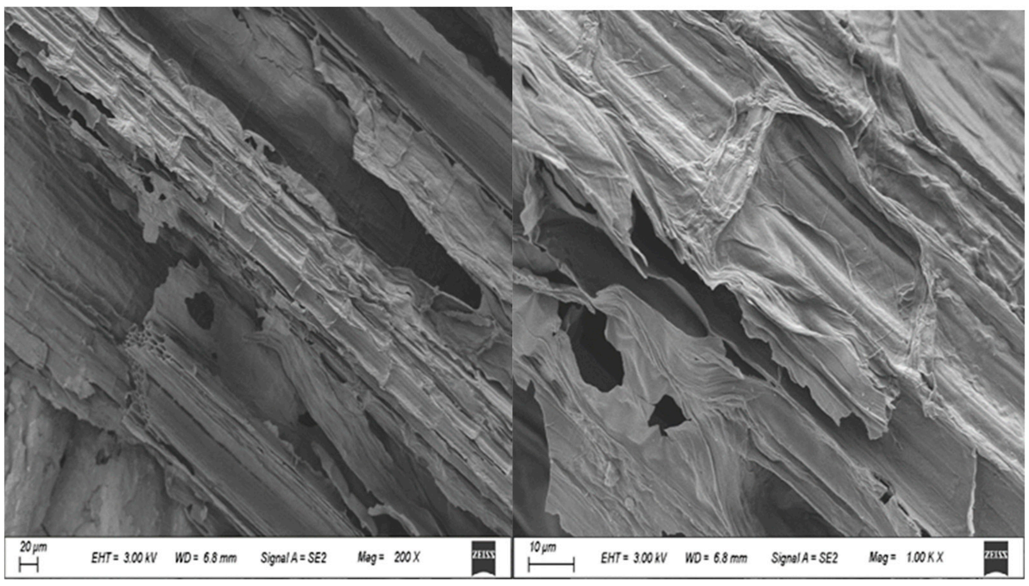


C

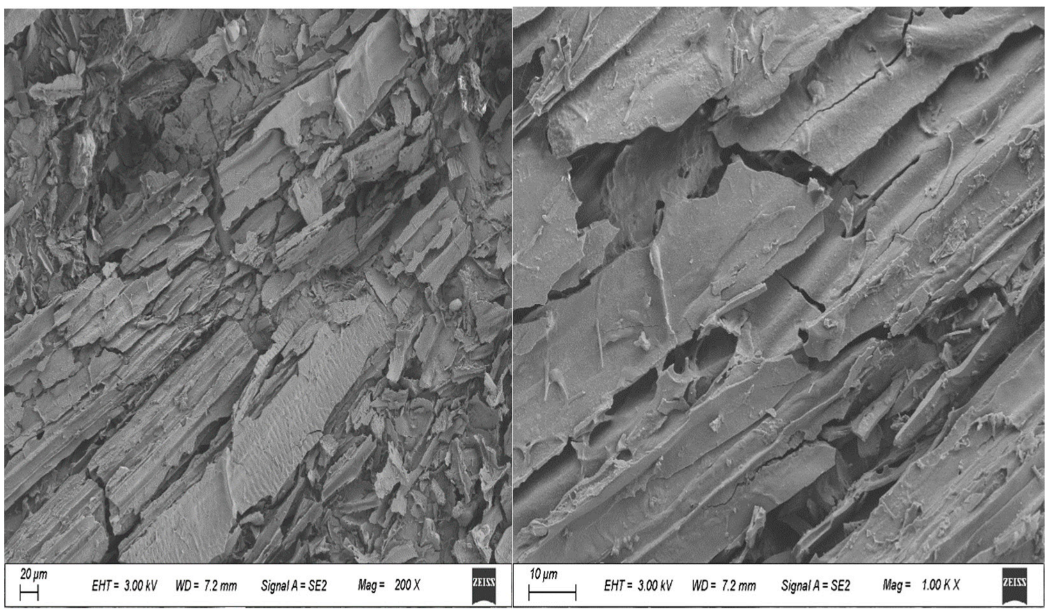


**Fig. S1.** Scanning Electron Microscope (SEM) images of A) MBC-300, B) MBC-400 and C) MBC-500 magnified 200 and 1000 times respectively.
